# Supplementary material for: Clinical factors affecting evoked magnetic fields in patients with Parkinson's disease
Source: PLoS One. 2020 Sep 17;15(9):e0232808. doi: 10.1371/journal.pone.0232808 (PMC7498017; doi:10.1371/journal.pone.0232808)
Supplement: S1 Table — Abbreviations: 1COMT, catechol-O-methyltransferase; 2LED, levodopa equivalent dose. (DOCX) [file pone.0232808.s001.docx]

**S1 Table. Details of the medications taken by patients.**

|  | **average** | **± SD** | **Number of treated patients** |
| --- | --- | --- | --- |
| Levodopa/carbidopa (mg) | 380.8 | ± 199.5 | 13 |
| Levodopa/benserazide (mg) | 285.0 | ± 102.9 | 10 |
| COMT^a^ inhibitor (mg) | 450.0 | ± 231.2 | 14 |
| Ropinirole (mg) | 6.9 | ± 5.1 | 8 |
| Pramipexole (mg) | 2.9 | ± 1.5 | 5 |
| Rotigotine (mg) | 18.6 | ± 12.8 | 8 |
| Pergolide (μg) | 750.0 | ± 0.0 | 1 |
| Selegiline (mg) | 5.0 | ± 2.6 | 14 |
| Rasagiline (mg) | 1.0 | ± 0.0 | 1 |
| Istradefylline (mg) | 32.7 | ± 0.1 | 11 |
| Trihexyphenidyl (mg) | 4.2 | ± 1.5 | 6 |
| Zonisamide (mg) | 61.1 | ± 33.3 | 9 |
| Droxidopa (mg) | 400.0 | ± 200.0 | 3 |
| Apomorphine (mg) | 10.0 | ± 0.0 | 1 |
| Amantadine (mg) | 133.3 | ± 28.9 | 3 |
| LED^b^ (mg) | 860.2 | ± 500.9 |  |

Abbreviations: ^1^COMT: catechol-*O*-methyltransferase, ^2^LED:lLevodopa equivalent dose.
