# Supplementary material for: Clinical factors affecting evoked magnetic fields in patients with Parkinson's disease
Source: PLoS One. 2020 Sep 17;15(9):e0232808. doi: 10.1371/journal.pone.0232808 (PMC7498017; doi:10.1371/journal.pone.0232808)
Supplement: S2 Table — Data are given as median [Q1–Q3]. Abbreviations: 1UE: upper extremity, 2LE: lower extremity. (DOCX) [file pone.0232808.s002.docx]

**S2 Table. Scores on the Unified Parkinson’s Disease Rating Scale (UPDRS) by patients with Parkinson’s disease.**

|  | | | **ON/usual** | | **OFF** | |
| --- | --- | --- | --- | --- | --- | --- |
| **Part 1** | | | 1.5 | [1–3.25] |  |  |
| Intellectual impairment | | | 1 | [0–1] |  |  |
| Thought disorder | | | 0.5 | [0–1.25] |  |  |
| Depression | | | 0 | [0–0] |  |  |
| Motivation/initiative | | | 0 | [0–1] |  |  |
| **Part 2** | | | 7.5 | [5–11] | 17.5 | [12.5–24.5] |
| Speech | | | 1 | [0–1] | 2 | [1–2] |
| Salivation | | | 0 | [0–1] | 1 | [0–2] |
| Swallowing | | | 0 | [0–1] | 0.5 | [0–1] |
| Handwriting | | | 1 | [1–1] | 2 | [1–3] |
| Cutting food/handing utensils | | | 0.5 | [0–1] | 1 | [1–2] |
| Dressing | | | 1 | [0–1] | 1 | [1–2] |
| Hygiene | | | 0 | [0–1] | 1 | [1–2] |
| Turning in bed/adjusting bed clothes | | | 1 | [0–2] | 2 | [1–3] |
| Falling, unrelated to freezing | | | 0.5 | [0–1] | 1 | [0–2.25] |
| Freezing when walking | | | 0 | [0–0.25] | 2 | [0.75–2.25] |
| Walking | | | 1 | [0.75–2] | 2 | [1.75–3] |
| Tremor | | | 0 | [0–1] | 1 | [0–1] |
| Sensory complaints | | | 0 | [0–0] | 0 | [0–1.25] |
| **Part 3** | | | 19 | [12.75–24.75] | 33.5 | [26–44.75] |
| Speech | | | 1 | [0–2] | 1.5 | [1–2] |
| Facial Expression | | | 1 | [1–2] | 1 | [1–2.25] |
| Tremor at rest | | Face | 0 | [0–0] | 0 | [0-0] |
|  |  | LUE^1^ | 0 | [0–0] | 0 | [0-0.25] |
|  |  | RUE | 0 | [0–0] | 0 | [0-0.25] |
|  |  | LLE^2^ | 0 | [0–0] | 0 | [0-0] |
|  |  | RLE | 0 | [0–0] | 0 | [0-0.25] |
| Action or postural tremor | | LUE | 0 | [0–1] | 0 | [0-1] |
|  |  | RUE | 0 | [0–1] | 0 | [0-0.25] |
| Rigidity | | Neck | 1 | [1–2] | 1 | [0.75-2] |
|  |  | RUE | 1 | [1–1] | 1.5 | [1-2] |
|  |  | LUE | 1 | [1–1] | 2 | [2-2] |
|  |  | LLE | 1 | [1–2] | 2 | [1-2.5] |
|  |  | RLE | 1 | [0–2] | 2 | [1.75-2.25] |
| Finger taps | | L | 1 | [0–1] | 1.5 | [0.75-2] |
|  |  | R | 1 | [0–1] | 1.5 | [1-2] |
| Hand movements | | L | 1 | [0–1] | 1.5 | [0.75-2] |
|  |  | R | 0.5 | [0–1] | 1 | [0.75-1.25] |
| Rapid alternating movements | | L | 1 | [0–2] | 1 | [0.75-2.5] |
|  |  | R | 1 | [0–1] | 1 | [0.75-2] |
| Leg agility | | L | 0 | [0–1] | 1 | [0.75-3] |
|  |  | R | 1 | [0–1] | 1 | [0.75-3] |
| Rising from chair | | | 0 | [0–0] | 1.5 | [0.75–4] |
| Posture | | | 1 | [1–2] | 1.5 | [0.75–2] |
| Gait | | | 1 | [0.75–1] | 2.5 | [1–3.25] |
| Postural stability | | | 1 | [0–2] | 2 | [2–4] |
| Body bradykinesia | | | 1 | [1–2] | 3 | [1.75–3.25] |
| **Part 4** | | | 4.5 | [3–8] |  |  |
| Dyskinesias | Duration | | 1 | [0–1] |  |  |
|  | Disability | | 0 | [0–0.25] |  |  |
|  | Painful dyskinesias | | 0 | [0–0] |  |  |
|  | Early morning dystonia | | 0 | [0–1] |  |  |
| Off | Predictable off | | 1 | [1–1] |  |  |
|  | Unpredictable off | | 0 | [0–1] |  |  |
|  | Sudden short off | | 0 | [0–1] |  |  |
|  | Total off time | | 1 | [1–2] |  |  |
| Anorexia, nausea, vomiting | | | 0 | [0–0] |  |  |
| Sleep disturbances | | | 0 | [0–1] |  |  |
| Symptomatic orthostasis | | | 0 | [0–1] |  |  |

Data are given as median [Q1–Q3]. Abbreviations: ^1^UE: upper extremity, ^2^LE: lower extremity
