# Supplementary material for: Clinical factors affecting evoked magnetic fields in patients with Parkinson's disease
Source: PLoS One. 2020 Sep 17;15(9):e0232808. doi: 10.1371/journal.pone.0232808 (PMC7498017; doi:10.1371/journal.pone.0232808)
Supplement: S3 Table — A comparison of the channel numbers of the sensor from which the maximum root mean square peak of each evoked field was derived. Each channel was subjected to the Wilcoxon signed-rank test as an order variable. Abbreviations: 1VEF: visual evoked magnetic field, 2AEF: auditory evoked magnetic field, 3SEF: somatosensory evoked field, 4p: p value. (DOCX) [file pone.0232808.s003.docx]

**S3 Table. Differences in individual site locations between patients with Parkinson’s disease and healthy control participants.**

|  | VEF^1^  N75m | | VEF  P100m | | VEF  N145m | | AEF^2^  P50m | | | | AEF  P100m | | | | SEF^3^  N20m | | SEF  P35m | | SEF  P60m | |
| --- | --- | --- | --- | --- | --- | --- | --- | --- | --- | --- | --- | --- | --- | --- | --- | --- | --- | --- | --- | --- |
|  | L | R | L | R | L | R | Li | Rc | Lc | Ri | Li | Rc | Lc | Ri | L | R | L | R | L | R |
| Patients | 164 | 172 | 171 | 164 | 163 | 161 | 24 | 24 |  | 132 | 24 | 24 | 122 | 132 | 113 | 44 | 113 | 181 | 113 | 23 |
|  | 171 | 173 | 203 | 171 | 193 | 171 | 151 | 152 | 261 | 261 | 151 | 151 | 261 | 261 | 241 | 162 | 241 | 23 | 242 | 162 |
|  | 173 | 173 | 203 | 172 | 203 | 173 | 24 | 24 |  |  | 24 | 24 | 122 | 132 | 113 | 44 | 222 | 181 | 222 | 182 |
|  | 174 | 174 | 203 | 192 | 203 | 174 | 24 | 24 | 132 | 144 | 24 | 24 | 132 | 132 | 113 | 44 | 113 | 44 | 114 | 43 |
|  | 174 | 192 | 211 | 192 | 203 | 181 | 162 | 151 | 264 | 264 | 24 | 24 | 252 | 261 | 241 | 164 | 241 | 181 | 133 | 23 |
|  | 181 | 193 | 211 | 192 | 211 | 192 | 24 | 24 | 133 | 133 | 24 | 24 | 133 | 133 | 241 | 181 | 241 | 181 | 241 | 181 |
|  | 193 | 193 | 211 | 192 | 212 | 192 | 154 | 151 | 262 | 144 | 14 | 154 | 143 | 144 | 133 | 151 | 264 | 12 | 261 | 161 |
|  | 203 | 194 | 212 | 192 | 221 | 194 | 24 | 24 | 261 | 132 | 24 | 24 | 132 | 132 | 241 | 181 | 222 | 12 | 241 | 44 |
|  | 212 | 204 | 212 | 192 | 222 | 204 | 24 | 24 | 261 | 133 | 21 | 24 | 261 | 261 | 113 | 44 | 113 | 43 | 222 | 44 |
|  | 212 | 204 | 212 | 193 | 231 | 211 | 24 | 161 | 133 | 133 | 24 | 162 | 133 | 133 | 113 | 181 | 244 | 43 | 222 | 181 |
|  | 212 | 211 | 231 | 193 | 231 | 212 | 23 | 23 | 133 | 133 | 23 | 23 | 133 | 133 | 222 | 43 | 222 | 43 | 222 | 43 |
|  | 213 | 212 | 233 | 194 | 233 | 231 | 152 | 23 | 261 | 261 | 24 | 24 | 261 | 261 | 134 | 181 | 241 | 11 | 134 | 43 |
|  | 213 | 233 | 233 | 194 | 234 | 232 | 24 | 24 | 142 | 134 | 24 | 24 | 133 | 142 | 241 | 44 | 113 | 181 | 222 | 181 |
|  | 221 | 233 | 234 | 204 | 234 | 241 | 21 | 151 | 133 | 133 | 24 | 24 | 133 | 133 | 113 | 23 | 223 | 23 | 113 | 22 |
|  | 222 | 242 | 234 | 211 | 234 | 241 | 151 | 151 | 144 | 144 | 151 | 151 | 144 | 144 | 264 | 152 | 264 | 164 | 243 | 172 |
|  | 231 | 243 | 234 | 211 | 242 | 243 | 24 | 24 | 133 | 133 | 24 | 24 | 133 | 133 | 222 | 23 | 91 | 181 | 241 | 44 |
|  | 234 | 243 | 234 | 211 | 254 | 243 | 24 | 24 | 133 | 133 | 24 | 24 | 133 | 133 | 113 | 162 | 92 | 182 | 222 | 181 |
|  | 244 | 244 | 234 | 212 | 262 | 252 | 23 | 21 | 133 | 133 | 24 | 151 | 133 | 133 | 134 | 162 | 241 | 162 | 134 | 162 |
|  | 254 | 253 | 251 | 251 | 264 | 254 | 151 | 24 | 261 | 261 | 151 | 151 | 264 | 261 | 241 | 44 | 241 | 161 | 241 | 184 |
|  | 264 | 261 | 263 | 264 | 264 | 264 | 24 | 24 | 133 | 132 | 24 | 24 | 133 | 133 | 241 | 162 | 221 | 162 | 134 | 163 |
| Controls | 173 | 211 | 164 | 192 | 174 | 163 | 24 | 24 | 132 | 142 | 24 | 24 | 133 | 133 | 222 | 162 | 222 | 181 | 241 | 162 |
|  | 194 | 161 | 203 | 192 | 203 | 163 | 24 | 24 | 133 | 133 | 21 | 24 | 133 | 133 | 134 | 44 | 113 | 44 | 241 | 44 |
|  | 203 | 191 | 203 | 192 | 203 | 164 | 23 | 21 | 261 | 134 | 24 | 24 | 261 | 132 | 134 | 23 | 241 | 44 | 241 | 23 |
|  | 203 | 192 | 211 | 192 | 211 | 172 | 161 | 24 | 133 | 261 | 24 | 161 | 261 | 261 | 113 | 23 | 113 | 181 | 222 | 23 |
|  | 211 | 192 | 231 | 192 | 211 | 173 | 23 | 24 | 133 | 132 | 24 | 24 | 132 | 132 | 113 | 44 | 113 | 44 | 113 | 44 |
|  | 211 | 192 | 233 | 192 | 231 | 192 | 24 | 24 | 132 | 133 | 24 | 24 | 132 | 132 | 113 | 44 | 113 | 44 | 113 | 44 |
|  | 222 | 194 | 234 | 192 | 232 | 192 | 162 | 24 | 133 | 134 | 24 | 24 | 133 | 132 | 113 | 44 | 113 | 181 | 113 | 181 |
|  | 234 | 204 | 234 | 192 | 234 | 193 | 24 | 24 | 133 | 133 | 24 | 24 | 133 | 133 | 113 | 44 | 113 | 44 | 113 | 44 |
|  | 234 | 212 | 234 | 193 | 243 | 204 | 24 | 24 | 133 | 133 | 24 | 24 | 133 | 133 | 241 | 23 | 241 | 162 | 241 | 162 |
|  | 243 | 231 | 251 | 193 | 252 | 251 | 24 | 24 | 133 | 133 | 24 | 24 | 133 | 133 | 113 | 44 | 113 | 43 | 113 | 43 |
| p^4^ | 1.000 | 0.103 | 0.800 | 0.075 | 0.381 | 0.016* | 0.762 | 0.233 | 0.036* | 0.577 | 0.646 | 0.431 | 0.535 | 0.072 | 0.083 | 0.027* | 0.083 | 0.608 | 0.205 | 0.210 |

A comparison of the channel numbers of the sensor from which the maximum root mean square peak of each evoked field was derived. Each channel was subjected to the Wilcoxon signed-rank test as an order variable.

Abbreviations: ^1^VEF: visual evoked magnetic field, ^2^AEF: auditory evoked magnetic field, ^3^SEF: somatosensory evoked field, ^4^p: p value
