# Supplementary material for: Clinical factors affecting evoked magnetic fields in patients with Parkinson's disease
Source: PLoS One. 2020 Sep 17;15(9):e0232808. doi: 10.1371/journal.pone.0232808 (PMC7498017; doi:10.1371/journal.pone.0232808)
Supplement: S4 Table — Abbreviations: 1CAT: Clinical Assessment for Attention, 2ADT: auditory detection task, 3SEF: somatosensory evoked magnetic field, 4M: mild side stimulation, 5S: severe side stimulation, 6VCT: visual cancellation task, 7VEF: visual evoked magnetic field, 8AEF: auditory evoked magnetic field, 9i: ipsilateral side recording, 10c: contralateral side recording, 11UPDRS: Unified Parkinson's Disease Rating Scale, 12LE: lower extremity, 13UE: upper extremity. (DOCX) [file pone.0232808.s004.docx]

**S4 Table. Correlation coefficients between clinical characteristics and measures, and evoked fields.**

| **CAT^1^** |  |  | R | p | Bonferroni correction |
| --- | --- | --- | --- | --- | --- |
| ADT^2^ | Hit rate | SEF^3^ P60m (M^4^) | -0.66 | 0.003 | 0.429 |
|  |  | SEF P60m (L) | -0.70 | 0.001 | 0.159 |
|  |  | SEF P60m - N20m (L) | -0.68 | 0.002 | 0.270 |
|  |  | SEF P60m - P35m (L) | -0.74 | 0.000 | 0.066 |
|  |  | SEF P60m - N20m (M) | -0.61 | 0.007 | 0.971 |
|  |  | SEF P60m - P35m (M) | -0.68 | 0.002 | 0.238 |
|  | Correct answer rate | SEF P60m - P35m (S^5^) | -0.62 | 0.006 | 0.902 |
| VCT^6^ (character) | Time | VEF^7^ P100m (L) | 0.62 | 0.003 | 0.485 |
| **Medication** |  |  | R | p | Bonferroni correction |
| Levodopa/benserazide | | AEF^8^ P100m (Mi^9^) | 0.91 | 0.000 | 0.038* |
|  |  | AEF P100m (Mc^10^) | 0.70 | 0.023 | 3.265 |
|  |  | AEF P100m (Li) | 0.68 | 0.029 | 4.045 |
|  |  | AEF P100m - P50m (Mc) | 0.79 | 0.006 | 0.897 |
|  |  | AEF P100m (Rc) | 0.68 | 0.030 | 4.196 |
|  |  | AEF P100m (Ri) | 0.91 | 0.000 | 0.030* |
|  |  | AEF P100m (Si) | 0.69 | 0.027 | 3.799 |
|  |  | VEF N75m (M) | 0.74 | 0.015 | 2.059 |
| Rotigotine |  | AEF P50m (Rc) | 0.75 | 0.031 | 4.293 |
| Ropinirole |  | VEF N75m (L) | 0.71 | 0.050 | 6.942 |
| **UPDRS^11^ Part 2 on** |  |  | r_s_ | p | Bonferroni correction |
| Total score |  | VEF P100m (L) | 0.61 | 0.004 | 0.549 |
| Speech |  | SEF P60m (R) | 0.61 | 0.004 | 0.599 |
|  |  | SEF P60m - N20m (R) | 0.68 | 0.001 | 0.148 |
|  |  | SEF P60m - P35m (R) | 0.70 | 0.001 | 0.091 |
|  |  | SEF P60m (S) | 0.64 | 0.002 | 0.341 |
|  |  | SEF P60m - N20m (S) | 0.68 | 0.001 | 0.135 |
|  |  | SEF P60m - P35m (S) | 0.66 | 0.001 | 0.199 |
| Swallowing |  | VEF P100m (L) | 0.64 | 0.002 | 0.318 |
| **UPDRS Part 3 on** |  |  | r_s_ | p | Bonferroni correction |
| Speech |  | AEF P100m (Ri) | 0.72 | 0.000 | 0.049* |
| Gait |  | VEF P100m (L) | 0.61 | 0.004 | 0.551 |
| **UPDRS part 3 off** |  |  | r_s_ | p | Bonferroni correction |
| Total score |  | AEF P100m (Mi) | 0.78 | 0.023 | 3.179 |
| Speech |  | VEF N75m (M) | -0.87 | 0.005 | 0.648 |
|  |  | VEF N75m (L) | -0.76 | 0.027 | 3.808 |
| Facial expression |  | AEF P100m (Mc) | 0.83 | 0.011 | 1.504 |
|  |  | AEF P50m (Mi) | 0.82 | 0.012 | 1.632 |
|  |  | AEF P100m (Li) | 0.81 | 0.015 | 2.088 |
|  |  | AEF P50m (Li) | 0.82 | 0.012 | 1.632 |
|  |  | AEF P100m (Rc) | 0.83 | 0.011 | 1.504 |
|  |  | AEF P100m (Sc) | 0.87 | 0.005 | 0.754 |
|  |  | SEF P60m (R) | 0.82 | 0.012 | 1.632 |
|  |  | SEF P60m (S) | 0.81 | 0.015 | 2.088 |
|  |  | SEF P60m - N20m (S) | 0.82 | 0.012 | 1.632 |
|  |  | SEF P60m - P35m (S) | 0.77 | 0.026 | 3.641 |
| Rigidity | LLE^12^ | SEF P60m (R) | 0.78 | 0.023 | 3.184 |
|  |  | VEF N75m (R) | -0.78 | 0.023 | 3.184 |
|  | RLE | SEF P60m (R) | 0.76 | 0.028 | 3.933 |
|  | SLE | AEF P100m (Mi) | 0.86 | 0.006 | 0.887 |
|  |  | SEF P60m (S) | 0.79 | 0.020 | 2.719 |
|  |  | SEF P60m - N20m (S) | 0.76 | 0.028 | 3.930 |
|  |  | SEF P60m - P35m (S) | 0.73 | 0.040 | 5.609 |
|  | MLE | AEF P100m (Mi) | 0.94 | 0.001 | 0.080 |
| Tremor at rest | LUE^13^ | VEF N75m (L) | -0.76 | 0.027 | 3.808 |
|  | RUE | VEF N75m (L) | -0.76 | 0.027 | 3.808 |
|  | SLE | VEF N75m (M) | -0.76 | 0.027 | 3.808 |
| Action or postural tremor | L | VEF P100m (L) | 0.73 | 0.039 | 5.390 |
|  |  | VEF N145m (L) | 0.74 | 0.037 | 5.147 |
|  | R | VEF P100m (L) | 0.76 | 0.030 | 4.173 |
|  |  | VEF N145m (L) | 0.76 | 0.028 | 3.959 |
| Finger taps | R | VEF P100m (L) | -0.81 | 0.015 | 2.102 |
|  |  | VEF N145m (L) | -0.81 | 0.014 | 1.954 |
|  | S | VEF N75m (M) | -0.87 | 0.005 | 0.716 |
|  | MLE | VEF N75m (M) | -0.75 | 0.031 | 4.293 |
| Hand movements | S | VEF N75m (M) | -0.86 | 0.006 | 0.774 |
|  | M | VEF N75m (M) | -0.83 | 0.011 | 1.495 |
| Rapid alternating movements | L | AEF P100m (Li) | 0.73 | 0.041 | 5.698 |
|  | S | AEF P100m (Si) | 0.87 | 0.005 | 0.706 |
|  | M | AEF P50m (Mi) | 0.80 | 0.017 | 2.346 |
|  |  | AEF P100m (Sc) | 0.83 | 0.012 | 1.599 |
| Leg agility | L | VEF N75m (R) | -0.80 | 0.016 | 2.279 |
|  | R | VEF N75m (R) | -0.80 | 0.016 | 2.279 |
|  | S | AEF P100m (Mi) | 0.78 | 0.023 | 3.247 |
|  | M | AEF P100m (Mi) | 0.78 | 0.023 | 3.247 |
| Arising from chair |  | AEF P100m (Li) | 0.76 | 0.029 | 4.068 |
|  |  | SEF P60m (R) | 0.82 | 0.014 | 1.895 |
|  |  | SEF P60m (S) | 0.75 | 0.034 | 4.680 |
|  |  | SEF P60m - N20m (S) | 0.74 | 0.035 | 4.912 |
| Posture |  | AEF P100m (Li) | 0.75 | 0.034 | 4.680 |
|  |  | SEF P60m (R) | 0.84 | 0.009 | 1.256 |
|  |  | SEF P60m (S) | 0.85 | 0.008 | 1.146 |
|  |  | SEF P60m - N20m (S) | 0.86 | 0.006 | 0.774 |
|  |  | SEF P60m - P35m (S) | 0.87 | 0.005 | 0.691 |
|  |  | VEF P100m (L) | 0.74 | 0.035 | 4.912 |
|  |  | VEF N145m (L) | 0.72 | 0.044 | 6.065 |
| Gait |  | AEF P100m (Mc) | 0.73 | 0.039 | 5.430 |
|  |  | AEF P50m (Mi) | 0.72 | 0.046 | 6.406 |
|  |  | AEF P100m (Li) | 0.77 | 0.026 | 3.604 |
|  |  | AEF P50m (Li) | 0.72 | 0.046 | 6.406 |
|  |  | AEF P100m (Si) | 0.72 | 0.045 | 6.250 |
|  |  | SEF P60m (R) | 0.82 | 0.012 | 1.643 |
|  |  | SEF P60m (S) | 0.88 | 0.004 | 0.573 |
|  |  | SEF P60m - N20m (S) | 0.90 | 0.003 | 0.350 |
|  |  | SEF P60m - P35m (S) | 0.88 | 0.004 | 0.573 |
| Postural stability |  | AEF P100m (Mi) | 0.72 | 0.046 | 6.423 |
|  |  | AEF P100m (Li) | 0.72 | 0.043 | 6.022 |
|  |  | AEF P100m (Rc) | 0.74 | 0.035 | 4.913 |
|  |  | AEF P100m (Sc) | 0.72 | 0.045 | 6.283 |
|  |  | SEF P60m (R) | 0.85 | 0.008 | 1.092 |
|  |  | SEF P60m (S) | 0.72 | 0.043 | 6.022 |
|  |  | SEF P60m - N20m (S) | 0.72 | 0.045 | 6.283 |
| Body bradykinesia |  | AEF P100m (Mi) | 0.80 | 0.017 | 2.325 |
|  |  | AEF P100m (Li) | 0.74 | 0.036 | 5.006 |
|  |  | SEF P60m (R) | 0.72 | 0.046 | 6.327 |
|  |  | SEF P60m (S) | 0.83 | 0.011 | 1.587 |
|  |  | SEF P60m - N20m (S) | 0.79 | 0.019 | 2.708 |
|  |  | SEF P60m - P35m (S) | 0.76 | 0.027 | 3.782 |
|  |  | VEF N75m (R) | -0.82 | 0.014 | 1.895 |
| **UPDRS off total** |  |  | r_s_ | p | Bonferroni correction |
| Total score |  | AEF P100m (Mi) | 0.75 | 0.031 | 4.241 |
|  |  | AEF P50m (Mi) | 0.71 | 0.047 | 6.467 |
|  |  | AEF P100m (Li) | 0.78 | 0.023 | 3.179 |
|  |  | AEF P50m (Li) | 0.71 | 0.047 | 6.467 |
|  |  | SEF P60m (R) | 0.76 | 0.028 | 3.893 |
|  |  | SEF P60m (S) | 0.83 | 0.011 | 1.591 |
|  |  | SEF P60m - N20m (S) | 0.81 | 0.015 | 2.071 |
|  |  | SEF P60m - P35m (S) | 0.77 | 0.027 | 3.686 |

Abbreviations: ^1^CAT: Clinical Assessment for Attention, ^2^ADT: auditory detection task, ^3^SEF: somatosensory evoked magnetic field, ^4^M: mild side stimulation, ^5^S: severe side stimulation, ^6^VCT: visual cancellation task, ^7^VEF: visual evoked magnetic field, ^8^AEF: auditory evoked magnetic field, ^9^i: ipsilateral side recording, ^10^c: contralateral side recording, ^11^UPDRS: Unified Parkinson's Disease Rating Scale, ^12^LE: lower extremity, ^13^UE: upper extremity
